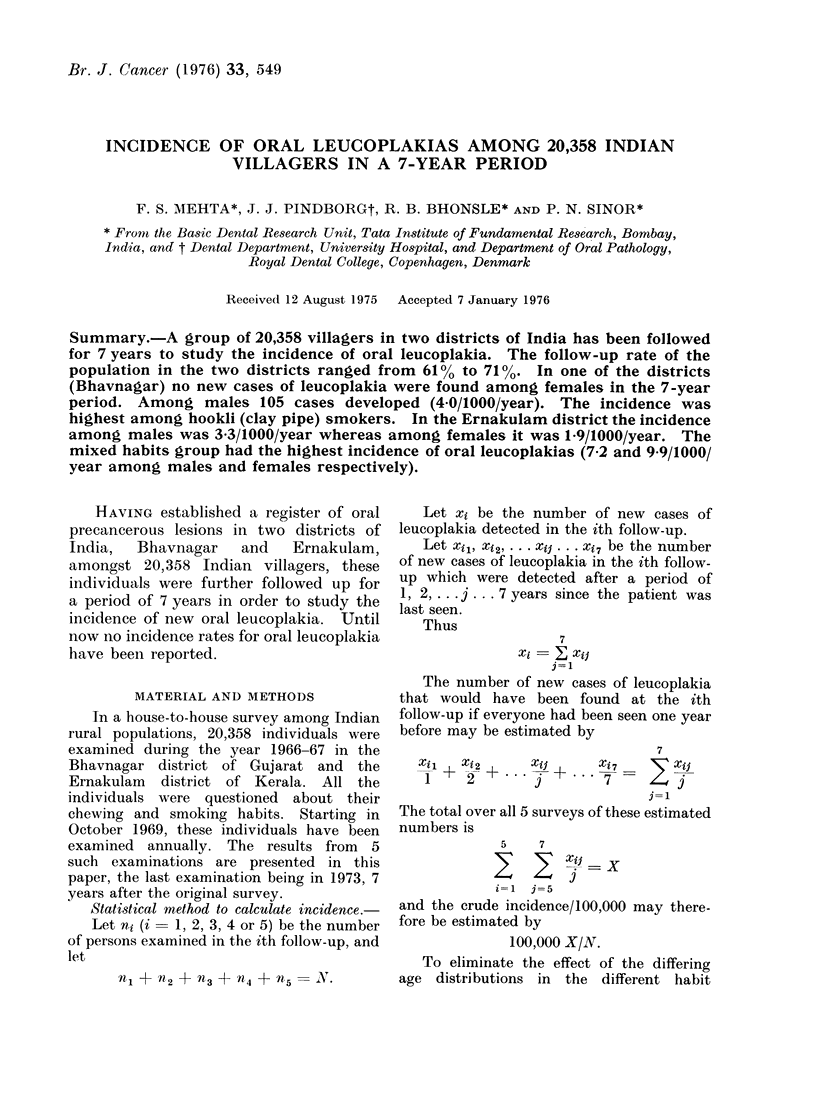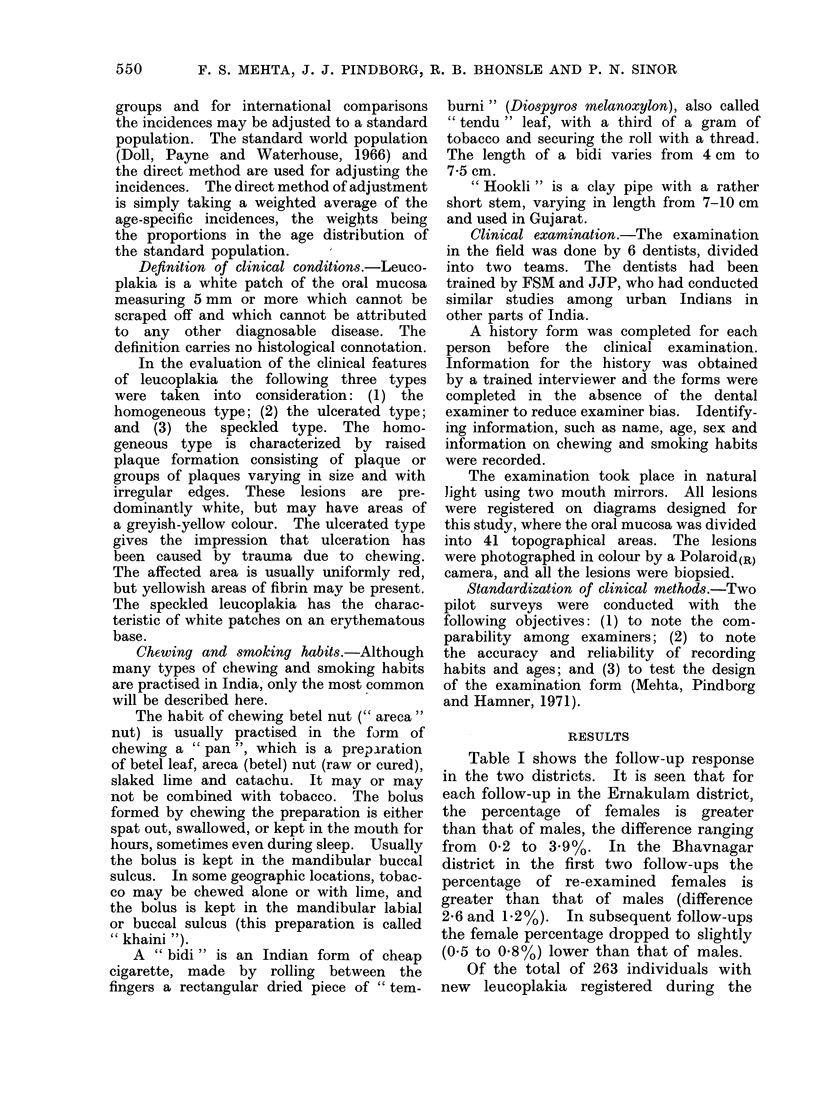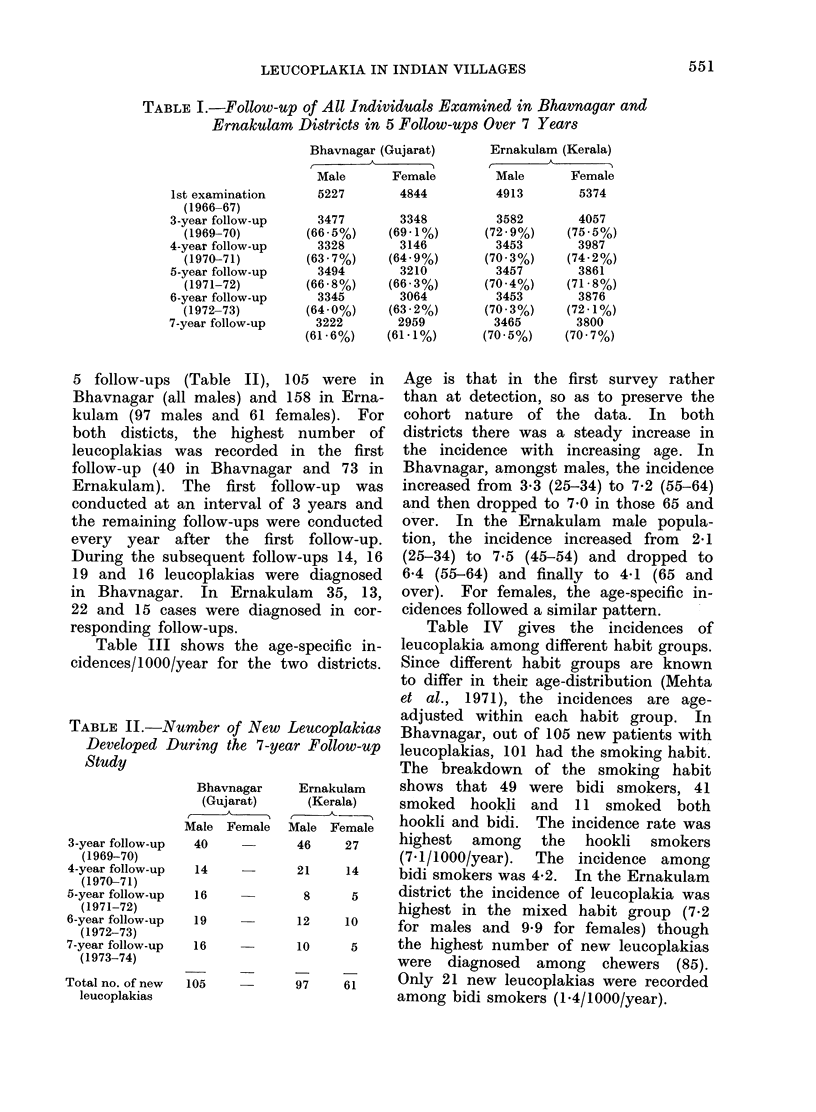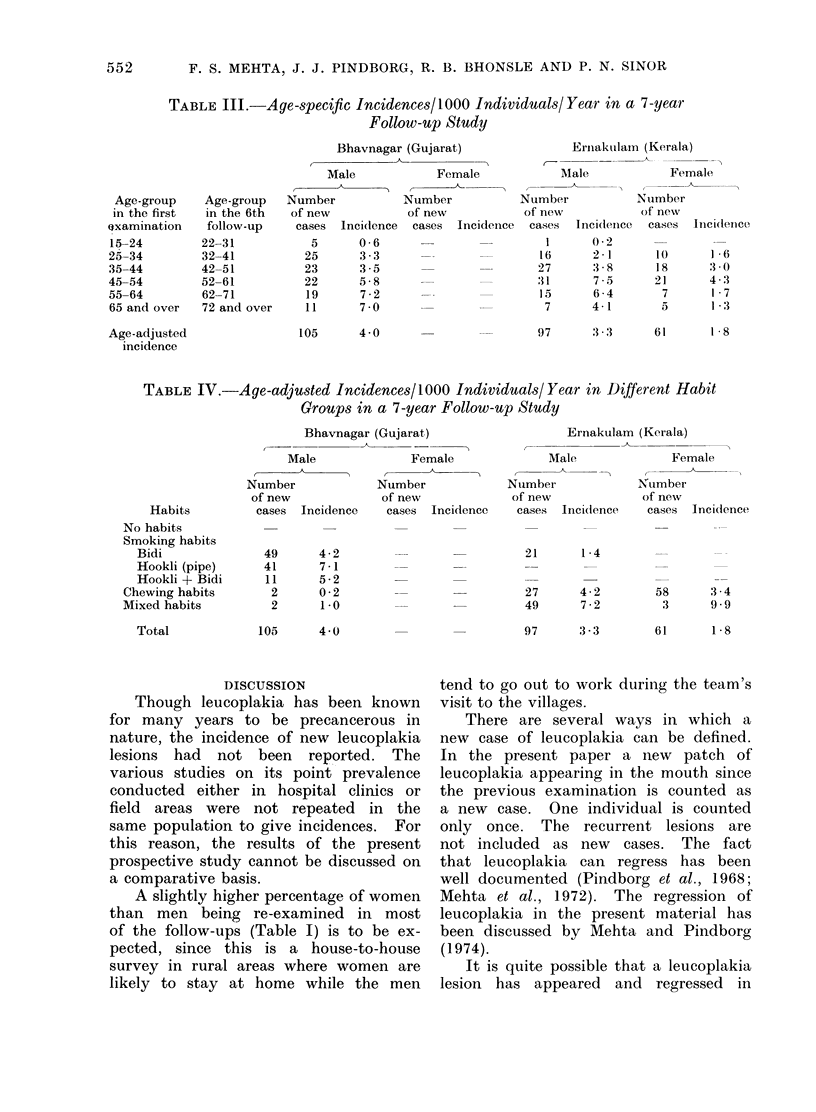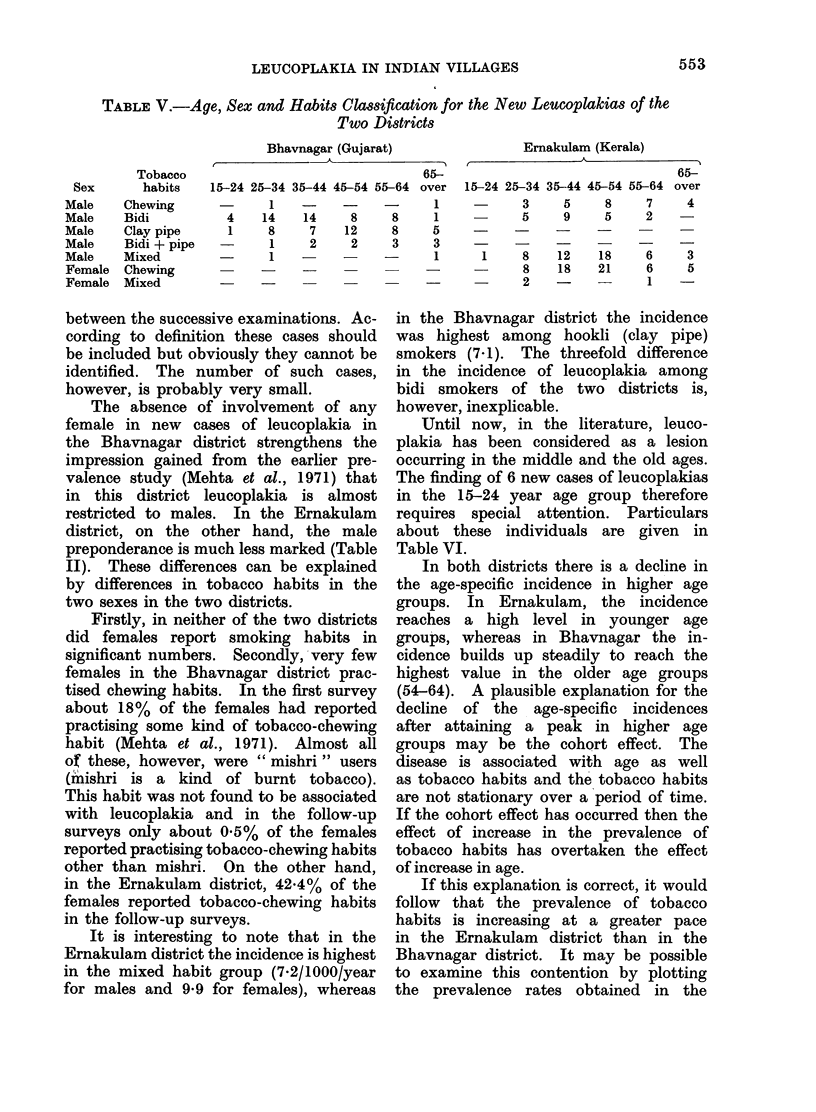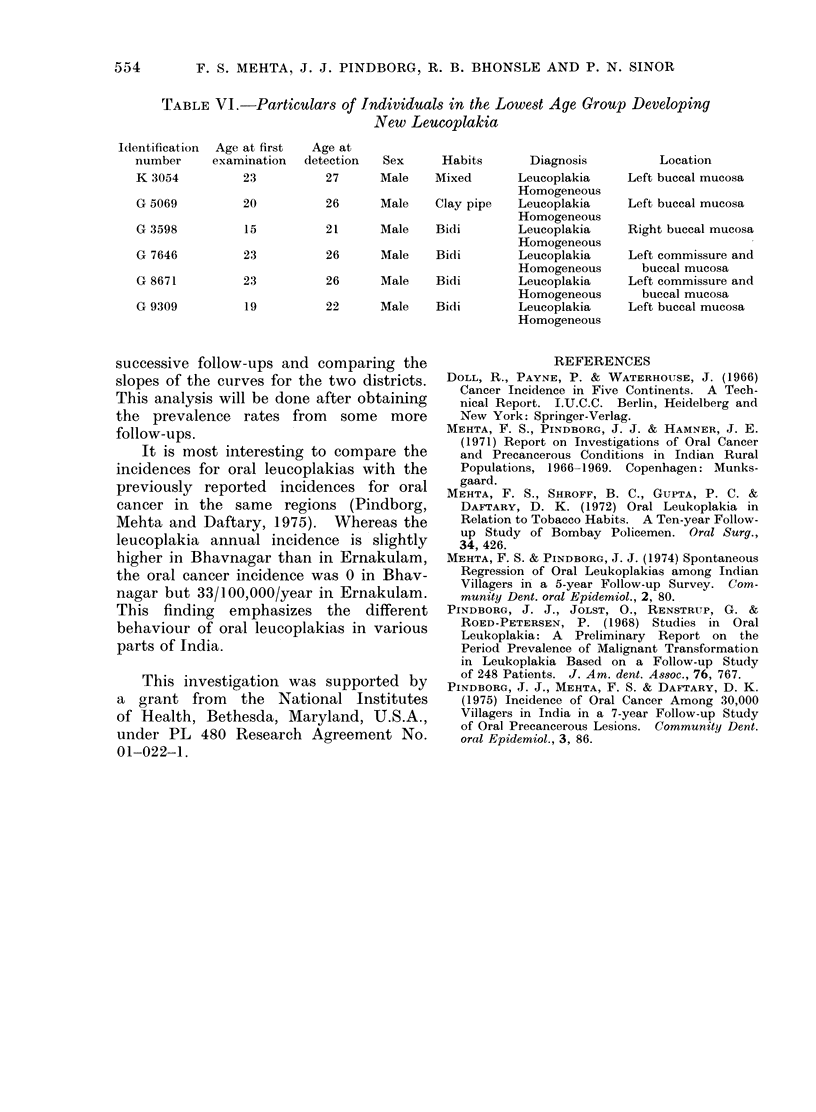# Incidence of oral leucoplakias among 20,358 Indian villagers in a 7-year period.

**DOI:** 10.1038/bjc.1976.87

**Published:** 1976-05

**Authors:** F. S. Mehta, J. J. Pindborg, R. B. Bhonsle, P. N. Sinor

## Abstract

A group of 20,358 villagers in two districts of India has been followed for 7 years to study the incidence of oral leucoplakia. The follow-up rate of the population in two districts ranged from 61% to 71%. In one of the districts (Bhavnagar) no new cases of leucoplakia were found among females in the 7-year period. Among males 105 cases developed (4-0/1000/year). The incidence was highest among hookli (clay pipe) smokers. In the Ernakulam district the incidence among males was 3-3/1000/year whereas among females it was 1-9/1000/year. The mixed habits group had the highest incidence of oral leucoplakias (7-2 and 9-9/1000/year among males and females respectively).


*Br. J. Cancer* (1976) **33**, 549

### INCIDENCE OF ORAL LEUCOPLAKIAS AMONG 20,358 INDIAN VILLAGERS IN A 7-YEAR PERIOD

F. S. MEHTA*, J. J. PINDBORG†, R. B. BHONSLE* AND P. N. SINOR*

* *From the Basic Dental Research Unit, Tata Institute of Fundamental Research, Bombay, India, and † Dental Department, University Hospital, and Department of Oral Pathology, Royal Dental College, Copenhagen, Denmark*

Received 12 August 1975 Accepted 7 January 1976

**Summary.—A group of 20,358 villagers in two districts of India has been followed for 7 years to study the incidence of oral leucoplakia. The follow-up rate of the population in the two districts ranged from 61% to 71%. In one of the districts (Bhavnagar) no new cases of leucoplakia were found among females in the 7-year period. Among males 105 cases developed (4·0/1000/year). The incidence was highest among hookli (clay pipe) smokers. In the Ernakulam district the incidence among males was 3·3/1000/year whereas among females it was 1·9/1000/year. The mixed habits group had the highest incidence of oral leucoplakias (7·2 and 9·9/1000/year among males and females respectively).**

HAVING established a register of oral precancerous lesions in two districts of India, Bhavnagar and Ernakulam, amongst 20,358 Indian villagers, these individuals were further followed up for a period of 7 years in order to study the incidence of new oral leucoplakia. Until now no incidence rates for oral leucoplakia have been reported.

### MATERIAL AND METHODS

In a house-to-house survey among Indian rural populations, 20,358 individuals were examined during the year 1966–67 in the Bhavnagar district of Gujarat and the Ernakulam district of Kerala. All the individuals were questioned about their chewing and smoking habits. Starting in October 1969, these individuals have been examined annually. The results from 5 such examinations are presented in this paper, the last examination being in 1973, 7 years after the original survey.

*Statistical method to calculate incidence.*—Let $n_i$ ($i = 1, 2, 3, 4$ or $5$) be the number of persons examined in the $i$th follow-up, and let

$$n_1 + n_2 + n_3 + n_4 + n_5 = N.$$

Let $x_i$ be the number of new cases of leucoplakia detected in the $i$th follow-up.

Let $x_{i1}, x_{i2}, \ldots x_{ij} \ldots x_{i7}$ be the number of new cases of leucoplakia in the $i$th follow-up which were detected after a period of $1, 2, \ldots j \ldots 7$ years since the patient was last seen.

Thus

$$x_i = \sum_{j=1}^{7} x_{ij}$$

The number of new cases of leucoplakia that would have been found at the $i$th follow-up if everyone had been seen one year before may be estimated by

$$\frac{x_{i1}}{1} + \frac{x_{i2}}{2} + \ldots \frac{x_{ij}}{j} + \ldots \frac{x_{i7}}{7} = \sum_{j=1}^{7} \frac{x_{ij}}{j}$$

The total over all 5 surveys of these estimated numbers is

$$\sum_{i=1}^{5} \sum_{j=5}^{7} \frac{x_{ij}}{j} = X$$

and the crude incidence/100,000 may therefore be estimated by

$$100{,}000\, X/N.$$

To eliminate the effect of the differing age distributions in the different habit groups and for international comparisons the incidences may be adjusted to a standard population. The standard world population (Doll, Payne and Waterhouse, 1966) and the direct method are used for adjusting the incidences. The direct method of adjustment is simply taking a weighted average of the age-specific incidences, the weights being the proportions in the age distribution of the standard population.

*Definition of clinical conditions.*—Leucoplakia is a white patch of the oral mucosa measuring 5 mm or more which cannot be scraped off and which cannot be attributed to any other diagnosable disease. The definition carries no histological connotation.

In the evaluation of the clinical features of leucoplakia the following three types were taken into consideration: (1) the homogeneous type; (2) the ulcerated type; and (3) the speckled type. The homogeneous type is characterized by raised plaque formation consisting of plaque or groups of plaques varying in size and with irregular edges. These lesions are predominantly white, but may have areas of a greyish-yellow colour. The ulcerated type gives the impression that ulceration has been caused by trauma due to chewing. The affected area is usually uniformly red, but yellowish areas of fibrin may be present. The speckled leucoplakia has the characteristic of white patches on an erythematous base.

*Chewing and smoking habits.*—Although many types of chewing and smoking habits are practised in India, only the most common will be described here.

The habit of chewing betel nut (" areca " nut) is usually practised in the form of chewing a " pan ", which is a preparation of betel leaf, areca (betel) nut (raw or cured), slaked lime and catachu. It may or may not be combined with tobacco. The bolus formed by chewing the preparation is either spat out, swallowed, or kept in the mouth for hours, sometimes even during sleep. Usually the bolus is kept in the mandibular buccal sulcus. In some geographic locations, tobacco may be chewed alone or with lime, and the bolus is kept in the mandibular labial or buccal sulcus (this preparation is called " khaini ").

A " bidi " is an Indian form of cheap cigarette, made by rolling between the fingers a rectangular dried piece of " temburni " (*Diospyros melanoxylon*), also called " tendu " leaf, with a third of a gram of tobacco and securing the roll with a thread. The length of a bidi varies from 4 cm to 7·5 cm.

" Hookli " is a clay pipe with a rather short stem, varying in length from 7–10 cm and used in Gujarat.

*Clinical examination.*—The examination in the field was done by 6 dentists, divided into two teams. The dentists had been trained by FSM and JJP, who had conducted similar studies among urban Indians in other parts of India.

A history form was completed for each person before the clinical examination. Information for the history was obtained by a trained interviewer and the forms were completed in the absence of the dental examiner to reduce examiner bias. Identifying information, such as name, age, sex and information on chewing and smoking habits were recorded.

The examination took place in natural light using two mouth mirrors. All lesions were registered on diagrams designed for this study, where the oral mucosa was divided into 41 topographical areas. The lesions were photographed in colour by a Polaroid$_{(R)}$ camera, and all the lesions were biopsied.

*Standardization of clinical methods.*—Two pilot surveys were conducted with the following objectives: (1) to note the comparability among examiners; (2) to note the accuracy and reliability of recording habits and ages; and (3) to test the design of the examination form (Mehta, Pindborg and Hamner, 1971).

### RESULTS

Table I shows the follow-up response in the two districts. It is seen that for each follow-up in the Ernakulam district, the percentage of females is greater than that of males, the difference ranging from 0·2 to 3·9%. In the Bhavnagar district in the first two follow-ups the percentage of re-examined females is greater than that of males (difference 2·6 and 1·2%). In subsequent follow-ups the female percentage dropped to slightly (0·5 to 0·8%) lower than that of males.

Of the total of 263 individuals with new leucoplakia registered during the

TABLE I.—*Follow-up of All Individuals Examined in Bhavnagar and Ernakulam Districts in 5 Follow-ups Over 7 Years*

| | Bhavnagar (Gujarat) | | Ernakulam (Kerala) | |
|---|---|---|---|---|
| | Male | Female | Male | Female |
| 1st examination (1966–67) | 5227 | 4844 | 4913 | 5374 |
| 3-year follow-up (1969–70) | 3477 (66·5%) | 3348 (69·1%) | 3582 (72·9%) | 4057 (75·5%) |
| 4-year follow-up (1970–71) | 3328 (63·7%) | 3146 (64·9%) | 3453 (70·3%) | 3987 (74·2%) |
| 5-year follow-up (1971–72) | 3494 (66·8%) | 3210 (66·3%) | 3457 (70·4%) | 3861 (71·8%) |
| 6-year follow-up (1972–73) | 3345 (64·0%) | 3064 (63·2%) | 3453 (70·3%) | 3876 (72·1%) |
| 7-year follow-up | 3222 (61·6%) | 2959 (61·1%) | 3465 (70·5%) | 3800 (70·7%) |

5 follow-ups (Table II), 105 were in Bhavnagar (all males) and 158 in Ernakulam (97 males and 61 females). For both disticts, the highest number of leucoplakias was recorded in the first follow-up (40 in Bhavnagar and 73 in Ernakulam). The first follow-up was conducted at an interval of 3 years and the remaining follow-ups were conducted every year after the first follow-up. During the subsequent follow-ups 14, 16 19 and 16 leucoplakias were diagnosed in Bhavnagar. In Ernakulam 35, 13, 22 and 15 cases were diagnosed in corresponding follow-ups.

Table III shows the age-specific incidences/1000/year for the two districts.

TABLE II.—*Number of New Leucoplakias Developed During the 7-year Follow-up Study*

| | Bhavnagar (Gujarat) | | Ernakulam (Kerala) | |
|---|---|---|---|---|
| | Male | Female | Male | Female |
| 3-year follow-up (1969–70) | 40 | — | 46 | 27 |
| 4-year follow-up (1970–71) | 14 | — | 21 | 14 |
| 5-year follow-up (1971–72) | 16 | — | 8 | 5 |
| 6-year follow-up (1972–73) | 19 | — | 12 | 10 |
| 7-year follow-up (1973–74) | 16 | — | 10 | 5 |
| Total no. of new leucoplakias | 105 | — | 97 | 61 |

Age is that in the first survey rather than at detection, so as to preserve the cohort nature of the data. In both districts there was a steady increase in the incidence with increasing age. In Bhavnagar, amongst males, the incidence increased from 3·3 (25–34) to 7·2 (55–64) and then dropped to 7·0 in those 65 and over. In the Ernakulam male population, the incidence increased from 2·1 (25–34) to 7·5 (45–54) and dropped to 6·4 (55–64) and finally to 4·1 (65 and over). For females, the age-specific incidences followed a similar pattern.

Table IV gives the incidences of leucoplakia among different habit groups. Since different habit groups are known to differ in their age-distribution (Mehta *et al.*, 1971), the incidences are age-adjusted within each habit group. In Bhavnagar, out of 105 new patients with leucoplakias, 101 had the smoking habit. The breakdown of the smoking habit shows that 49 were bidi smokers, 41 smoked hookli and 11 smoked both hookli and bidi. The incidence rate was highest among the hookli smokers (7·1/1000/year). The incidence among bidi smokers was 4·2. In the Ernakulam district the incidence of leucoplakia was highest in the mixed habit group (7·2 for males and 9·9 for females) though the highest number of new leucoplakias were diagnosed among chewers (85). Only 21 new leucoplakias were recorded among bidi smokers (1·4/1000/year).

TABLE III.—*Age-specific Incidences/1000 Individuals/Year in a 7-year Follow-up Study*

| | | Bhavnagar (Gujarat) | | | | Ernakulam (Kerala) | | | |
|---|---|---|---|---|---|---|---|---|---|
| | | Male | | Female | | Male | | Female | |
| Age-group in the first examination | Age-group in the 6th follow-up | Number of new cases | Incidence | Number of new cases | Incidence | Number of new cases | Incidence | Number of new cases | Incidence |
| 15–24 | 22–31 | 5 | 0·6 | — | — | 1 | 0·2 | — | — |
| 25–34 | 32–41 | 25 | 3·3 | — | — | 16 | 2·1 | 10 | 1·6 |
| 35–44 | 42–51 | 23 | 3·5 | — | — | 27 | 3·8 | 18 | 3·0 |
| 45–54 | 52–61 | 22 | 5·8 | — | — | 31 | 7·5 | 21 | 4·3 |
| 55–64 | 62–71 | 19 | 7·2 | — | — | 15 | 6·4 | 7 | 1·7 |
| 65 and over | 72 and over | 11 | 7·0 | — | — | 7 | 4·1 | 5 | 1·3 |
| Age-adjusted incidence | | 105 | 4·0 | — | — | 97 | 3·3 | 61 | 1·8 |

TABLE IV.—*Age-adjusted Incidences/1000 Individuals/Year in Different Habit Groups in a 7-year Follow-up Study*

| | Bhavnagar (Gujarat) | | | | Ernakulam (Kerala) | | | |
|---|---|---|---|---|---|---|---|---|
| | Male | | Female | | Male | | Female | |
| Habits | Number of new cases | Incidence | Number of new cases | Incidence | Number of new cases | Incidence | Number of new cases | Incidence |
| No habits | — | — | — | — | — | — | — | — |
| Smoking habits | | | | | | | | |
| Bidi | 49 | 4·2 | — | — | 21 | 1·4 | — | — |
| Hookli (pipe) | 41 | 7·1 | — | — | — | — | — | — |
| Hookli + Bidi | 11 | 5·2 | — | — | — | — | — | — |
| Chewing habits | 2 | 0·2 | — | — | 27 | 4·2 | 58 | 3·4 |
| Mixed habits | 2 | 1·0 | — | — | 49 | 7·2 | 3 | 9·9 |
| Total | 105 | 4·0 | — | — | 97 | 3·3 | 61 | 1·8 |

### DISCUSSION

Though leucoplakia has been known for many years to be precancerous in nature, the incidence of new leucoplakia lesions had not been reported. The various studies on its point prevalence conducted either in hospital clinics or field areas were not repeated in the same population to give incidences. For this reason, the results of the present prospective study cannot be discussed on a comparative basis.

A slightly higher percentage of women than men being re-examined in most of the follow-ups (Table I) is to be expected, since this is a house-to-house survey in rural areas where women are likely to stay at home while the men tend to go out to work during the team's visit to the villages.

There are several ways in which a new case of leucoplakia can be defined. In the present paper a new patch of leucoplakia appearing in the mouth since the previous examination is counted as a new case. One individual is counted only once. The recurrent lesions are not included as new cases. The fact that leucoplakia can regress has been well documented (Pindborg *et al.*, 1968; Mehta *et al.*, 1972). The regression of leucoplakia in the present material has been discussed by Mehta and Pindborg (1974).

It is quite possible that a leucoplakia lesion has appeared and regressed in

TABLE V.—*Age, Sex and Habits Classification for the New Leucoplakias of the Two Districts*

| | | Bhavnagar (Gujarat) | | | | | | Ernakulam (Kerala) | | | | | |
|---|---|---|---|---|---|---|---|---|---|---|---|---|---|
| Sex | Tobacco habits | 15–24 | 25–34 | 35–44 | 45–54 | 55–64 | 65–over | 15–24 | 25–34 | 35–44 | 45–54 | 55–64 | 65–over |
| Male | Chewing | — | 1 | — | — | — | 1 | — | 3 | 5 | 8 | 7 | 4 |
| Male | Bidi | 4 | 14 | 14 | 8 | 8 | 1 | — | 5 | 9 | 5 | 2 | — |
| Male | Clay pipe | 1 | 8 | 7 | 12 | 8 | 5 | — | — | — | — | — | — |
| Male | Bidi + pipe | — | 1 | 2 | 2 | 3 | 3 | — | — | — | — | — | — |
| Male | Mixed | — | 1 | — | — | — | 1 | 1 | 8 | 12 | 18 | 6 | 3 |
| Female | Chewing | — | — | — | — | — | — | — | 8 | 18 | 21 | 6 | 5 |
| Female | Mixed | — | — | — | — | — | — | — | 2 | — | — | 1 | — |

between the successive examinations. According to definition these cases should be included but obviously they cannot be identified. The number of such cases, however, is probably very small.

The absence of involvement of any female in new cases of leucoplakia in the Bhavnagar district strengthens the impression gained from the earlier prevalence study (Mehta *et al.*, 1971) that in this district leucoplakia is almost restricted to males. In the Ernakulam district, on the other hand, the male preponderance is much less marked (Table II). These differences can be explained by differences in tobacco habits in the two sexes in the two districts.

Firstly, in neither of the two districts did females report smoking habits in significant numbers. Secondly, very few females in the Bhavnagar district practised chewing habits. In the first survey about 18% of the females had reported practising some kind of tobacco-chewing habit (Mehta *et al.*, 1971). Almost all of these, however, were " mishri " users (mishri is a kind of burnt tobacco). This habit was not found to be associated with leucoplakia and in the follow-up surveys only about 0·5% of the females reported practising tobacco-chewing habits other than mishri. On the other hand, in the Ernakulam district, 42·4% of the females reported tobacco-chewing habits in the follow-up surveys.

It is interesting to note that in the Ernakulam district the incidence is highest in the mixed habit group (7·2/1000/year for males and 9·9 for females), whereas in the Bhavnagar district the incidence was highest among hookli (clay pipe) smokers (7·1). The threefold difference in the incidence of leucoplakia among bidi smokers of the two districts is, however, inexplicable.

Until now, in the literature, leucoplakia has been considered as a lesion occurring in the middle and the old ages. The finding of 6 new cases of leucoplakias in the 15–24 year age group therefore requires special attention. Particulars about these individuals are given in Table VI.

In both districts there is a decline in the age-specific incidence in higher age groups. In Ernakulam, the incidence reaches a high level in younger age groups, whereas in Bhavnagar the incidence builds up steadily to reach the highest value in the older age groups (54–64). A plausible explanation for the decline of the age-specific incidences after attaining a peak in higher age groups may be the cohort effect. The disease is associated with age as well as tobacco habits and the tobacco habits are not stationary over a period of time. If the cohort effect has occurred then the effect of increase in the prevalence of tobacco habits has overtaken the effect of increase in age.

If this explanation is correct, it would follow that the prevalence of tobacco habits is increasing at a greater pace in the Ernakulam district than in the Bhavnagar district. It may be possible to examine this contention by plotting the prevalence rates obtained in the

TABLE VI.—*Particulars of Individuals in the Lowest Age Group Developing New Leucoplakia*

| Identification number | Age at first examination | Age at detection | Sex | Habits | Diagnosis | Location |
|---|---|---|---|---|---|---|
| K 3054 | 23 | 27 | Male | Mixed | Leucoplakia Homogeneous | Left buccal mucosa |
| G 5069 | 20 | 26 | Male | Clay pipe | Leucoplakia Homogeneous | Left buccal mucosa |
| G 3598 | 15 | 21 | Male | Bidi | Leucoplakia Homogeneous | Right buccal mucosa |
| G 7646 | 23 | 26 | Male | Bidi | Leucoplakia Homogeneous | Left commissure and buccal mucosa |
| G 8671 | 23 | 26 | Male | Bidi | Leucoplakia Homogeneous | Left commissure and buccal mucosa |
| G 9309 | 19 | 22 | Male | Bidi | Leucoplakia Homogeneous | Left buccal mucosa |

successive follow-ups and comparing the slopes of the curves for the two districts. This analysis will be done after obtaining the prevalence rates from some more follow-ups.

It is most interesting to compare the incidences for oral leucoplakias with the previously reported incidences for oral cancer in the same regions (Pindborg, Mehta and Daftary, 1975). Whereas the leucoplakia annual incidence is slightly higher in Bhavnagar than in Ernakulam, the oral cancer incidence was 0 in Bhavnagar but 33/100,000/year in Ernakulam. This finding emphasizes the different behaviour of oral leucoplakias in various parts of India.

This investigation was supported by a grant from the National Institutes of Health, Bethesda, Maryland, U.S.A., under PL 480 Research Agreement No. 01–022–1.

### REFERENCES

DOLL, R., PAYNE, P. & WATERHOUSE, J. (1966) Cancer Incidence in Five Continents. A Technical Report. I.U.C.C. Berlin, Heidelberg and New York: Springer-Verlag.

MEHTA, F. S., PINDBORG, J. J. & HAMNER, J. E. (1971) Report on Investigations of Oral Cancer and Precancerous Conditions in Indian Rural Populations, 1966–1969. Copenhagen: Munksgaard.

MEHTA, F. S., SHROFF, B. C., GUPTA, P. C. & DAFTARY, D. K. (1972) Oral Leukoplakia in Relation to Tobacco Habits. A Ten-year Follow-up Study of Bombay Policemen. *Oral Surg.*, **34**, 426.

MEHTA, F. S. & PINDBORG, J. J. (1974) Spontaneous Regression of Oral Leukoplakias among Indian Villagers in a 5-year Follow-up Survey. *Community Dent. oral Epidemiol.*, **2**, 80.

PINDBORG, J. J., JOLST, O., RENSTRUP, G. & ROED-PETERSEN, P. (1968) Studies in Oral Leukoplakia: A Preliminary Report on the Period Prevalence of Malignant Transformation in Leukoplakia Based on a Follow-up Study of 248 Patients. *J. Am. dent. Assoc.*, **76**, 767.

PINDBORG, J. J., MEHTA, F. S. & DAFTARY, D. K. (1975) Incidence of Oral Cancer Among 30,000 Villagers in India in a 7-year Follow-up Study of Oral Precancerous Lesions. *Community Dent. oral Epidemiol.*, **3**, 86.